# Enhanced Signal-to-Noise and Fast Calibration of Optical Tweezers Using Single Trapping Events

**DOI:** 10.3390/mi12050570

**Published:** 2021-05-17

**Authors:** Alexander B. Stilgoe, Declan J. Armstrong, Halina Rubinsztein-Dunlop

**Affiliations:** 1 School of Mathematics and Physics, The University of Queensland, St. Lucia, Brisbane, QLD 4072, Australia; declan.armstrong@uq.net.au; 2 Australian Research Council Centre of Excellence for Engineered Quantum Systems, School of Mathematics and Physics, University of Queensland, St. Lucia, QLD 4072, Australia

**Keywords:** optical tweezers, optically driven swimmers, swimmers, swimming cells, manipulation, calibration

## Abstract

The trap stiffness us the key property in using optical tweezers as a force transducer. Force reconstruction via maximum-likelihood-estimator analysis (FORMA) determines the optical trap stiffness based on estimation of the particle velocity from statistical trajectories. Using a modification of this technique, we determine the trap stiffness for a two micron particle within 2 ms to a precision of ∼10% using camera measurements at 10 kfps with the contribution of pixel noise to the signal being larger the level Brownian motion. This is done by observing a particle fall into an optical trap once at a high stiffness. This type of calibration is attractive, as it avoids the use of a nanopositioning stage, which makes it ideal for systems of large numbers of particles, e.g., micro-fluidics or active matter systems.

## 1. Introduction

Optical tweezers are an important tool for measuring femto-to-nano newton forces in soft matter and biological systems [1,2,3]. The operating principle of optical tweezers is the transfer of momentum from highly focused light through deflection to confine a particle. A spherical particle will deflect highly focused light in much the same way a spherical lens would. For small displacements the transfer of momentum will be proportional to negative displacement, thus acting as a linear spring. A key challenge in these measurements is the calibration of the linear response. One of the most popular calibration methods is to fit the power spectral density of the trapped particles motion to that of an over damped harmonic oscillator model [4,5,6]. The technique is reasonably reliable as most optical tweezers display the necessary linear response to displacement. Active drive variations of calibration of linear models are particularly useful for making viscoelasticity measurements [7,8,9,10,11]. There are alternatives to power spectrum calibration, including Bayesian inference [12,13], which does not depend on input parameters and it is also less sensitive to systematic errors. It infers the diffusion coefficient and the potential felt by a bead trapped optically. It uses comparatively larger amount of the information stored in the recorded bead trajectory than the standard calibration approaches. Another alternative method is force reconstruction via maximum likelihood estimation (FORMA) [14], in which one retrieves the force field acting on a Brownian particle from the analysis of its observed motion within the macroscopic force field. FORMA is an attractive alternative to many of the other techniques, as it only consists of determining the ratio of two sums that arise from analysis of the occupation probability within the trap.

When the calibration is done for increasing trap stiffness a number of corrections need to be made as compared to the lower trap stiffness to account for aliasing and blurring that can significantly change the prediction of trapping force from camera calibrations [15,16,17,18]. The signal-to-noise is too low to perform calibrations shorter than about one second at equilibrium. In microfluidics and active matter systems, large numbers of objects are continually moving through the system, such that time resolutions are insufficient for investigating the forces acting on these particles. We are going to use a variation of FORMA that takes aliasing into account for the purposes of rapid calibration of an optical tweezers and assess its accuracy. Including position measurement uncertainty of about 10 nm, we will demonstrate that a trap can be calibrated to about ∼10% precision for micron sized particles that fall into the trap once. For contrast, the Brownian wandering distance for a 2 μm particle is about 7 nm over 100 μs. This approach takes advantage of the improved signal-to-noise as the particle falls into the center of the trap. In concept, this resembles blinking tweezers schemes, where particles are driven out of the trap to fall in and improve the signal-to-noise ratio of the measurements [8,19,20,21]. The high-bandwidth noise that is introduced by Brownian motion is suppressed. We show, in an experiment, that single descents can be used to calibrate the optical trap where large trap strengths restrict the performance of equilibrium measurements as they are in a regime where the signal is too close to the detection noise floor. This type of calibration is ideal for multiple and dynamic optical traps to rapidly determine the swimming forces of optically driven micromachines, active matter, and cells on the two micron size scale [22,23,24,25,26,27,28].

### Theoretical Framework

Optical tweezers are simply modelled as an over-damped linear spring with the differential equation $\dot{x}\left(t\right)=\omega_{c}x\left(t\right)$, where $\omega_{c}$ is the linear response constant or angular trap frequency. If this linear system exists in the presence of normally distributed noise, ξ(t), and with $\omega_{c}<0$, then the position of the particle will have the following continuous probability distribution function:

(1) $$P\left(x;\sigma_{x}^{2}\right)=\frac{1}{\sqrt{2\pi \sigma_{x}^{2}}}e^{-\frac{x^{2}}{2\sigma_{x}^{2}}},$$

with likelihood, $L\left(x;\sigma_{x}^{2}\right)=\prod_{n}P\left(x_{n};\sigma_{x}^{2}\right)$ where $x_{n}$ is the position of the particle at event number *n* and overall variance $\sigma_{x}^{2}$. The probability distribution for the mean regressed linear response problem is given by

(2) $$P\left(x,y;\omega ,\sigma^{2}\right)=\frac{1}{\sqrt{2\pi \sigma^{2}}}e^{-\frac{{\left(y/\Delta t-\omega x\right)}^{2}}{2\sigma^{2}}},$$

where we have set the estimator to have the property y/Δt→dx/dt as Δt→0. Finding the maximum of the natural logarithm of the resulting likelihood function with respect to ω (∂logL/∂ω) we obtain the key result of FORMA [14] for 1D:

(3) $$\omega =\frac{1}{\Delta t}\frac{\sum_{n}x_{n}y_{n}}{\sum_{n}x_{n}^{2}}≃\omega_{c},$$

where the difference in position at two sequential time-steps is $y_{n}=x_{n+1}-x_{n}$. We have assumed that $y_{n}/\Delta t$ is the estimator of velocity (and thus $\omega =\omega_{c}$), but this is not exactly the case. A more accurate approximation based on the finite time difference of the linear model is:

(4) $$\begin{array}{cc} \xi_{n} & =y_{n}-x_{n}\left(e^{\omega_{c}\Delta t}-1\right),\\ \; & =x_{n+1}-e^{\omega_{c}\Delta t}x_{n}, \end{array}$$

where $\omega_{c}$ is the angular trap frequency and $\xi_{n}$ is the appropriate discrete noise for the system. The corrected angular trap frequency is now:

(5) $$\omega_{\mathrm{corr}}=\frac{\log\left(1+\frac{\sum_{n}x_{n}y_{n}}{\sum_{n}x_{n}^{2}}\right)}{\Delta t}=\frac{\log\frac{\sum_{n}x_{n}x_{n+1}}{\sum_{n}x_{n}^{2}}}{\Delta t}.$$

The expression remains valid while $\frac{\sum_{n}x_{n}x_{n+1}}{\sum_{n}x_{n}^{2}}>0$ (which becomes more likely to be statistically violated when the actual angular trap frequency $\omega_{c}<-2\pi /\sqrt{10}\Delta t$, and equipartition calibration should be used instead). This correction is in practice equivalent to accounting for aliasing in a power spectrum calibration, as shown in [5]. We can take account of the discrete sampling. The measurements are a snap shot of the experiment at discrete times. Assuming that the properties of the noise do not change over each time step we correct the FORMA diffusion constant by considering that the discrete noise $\xi_{n}$ is the cumulative result of a continuous noise source within a linear system over the time interval. Thus, we estimate the correction to variance, *c*, using an Itô integral [5,29] over each time step and the estimated aliasing compensated diffusion constant of FORMA is then:

(6) $$\begin{array}{cc} D_{\mathrm{est}} & =\frac{c}{2\Delta t}\frac{1}{N}\sum_{n}^{N}{\left(x_{n+1}-e^{\omega_{\mathrm{corr}}\Delta t}x_{n}\right)}^{2},\\ \; & =\frac{1}{2\Delta t}\frac{2\omega_{\mathrm{corr}}\Delta t}{e^{2\omega_{\mathrm{corr}}\Delta t}-1}\frac{1}{N}\sum_{n}^{N}{\left(x_{n+1}-e^{\omega_{\mathrm{corr}}\Delta t}x_{n}\right)}^{2}, \end{array}$$

which gives good agreement for the parameter range that $\omega_{\mathrm{corr}}$ is an accurate angular trap frequency, but it will begin to diverge at frequencies outside the stable region where the fringe high variance events limit the accuracy of the method.

The effect of blurring from taking camera images is altogether a different matter. Under normal trap conditions, i.e., at the equilibrium, the effect of blurring is large [18]. More critically, the effect of blur is magnified in the velocity spectrum, which increases the difficulty of using FORMA, as it is the convolution of the continuous time series with a rect function. Fortunately, because a particle falling into the trap is on an exponential trajectory, the result of the blurring is to change the observed position of the particle, but not the relationship between the position and velocity estimator. Thus, the measured angular trap frequency is unaffected by blur in our particular case, but it will affect any estimate of the diffusion constant for the particle.

## 2. Materials and Methods

### 2.1. Simulation

To characterise the convergence of the fit, we performed numerical simulations of a particle undergoing Brownian motion in an ideal harmonic oscillator using second order Runge–Kutta (RK2) intermediate integration time steps [30]. To reduce the discretisation error, the simulation was performed with time steps of $\Delta t=10^{-5}$s and down sampled to 10 kHz for analysis. To assess the performance of the aliasing compensated FORMA, we simulated data for 2 μm diameter particles.

**Figure 1:**
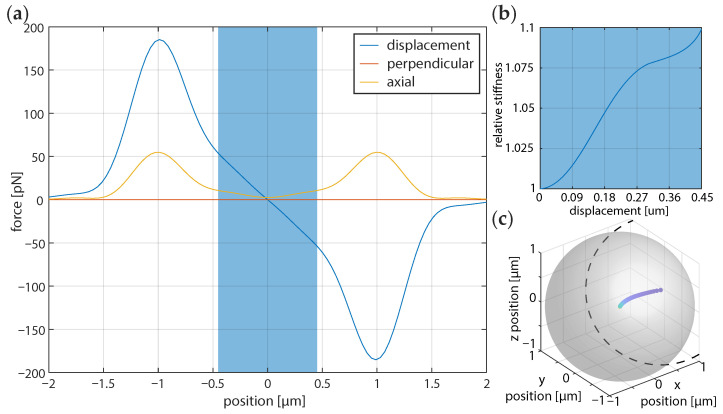
Calculation of the force extension curves for the optical force acting on particles of radius 1.0 μm for displacement along one axis in the beam focal plane. (**a**) The approximately linear region is marked by the shaded rectangle. (**b**) The approximate stiffness (initial force divided by displacement from equilibrium) of the optical trap in the shaded region relative to the equilibrium value. (**c**) Trajectory for such a particle from a displacement of 1 μm along one of the transverse axes with an overlaid shaded region representing the outer surface of the sphere. It moves along the beam axis due to scattering forces.

A particle falling into an optical trap can be analysed with FORMA when the trap exhibits a linear response to displacement. To this end, we characterised the linear region for polystyrene particles with a size of 2 μm in diameter using the optical tweezers toolbox [31] (Downloadable at: https://github.com/ilent2/ott (accessed on 1 March 2021)). The calculation is setup for trapping of a 2 μm diameter spherical particle with refractive index n=1.57 in water with a N.A. 1.2 objective with linear `x’ polarised light with a Gaussian profile truncated at the aperture. Figure 1 shows the optical force on these particles as a function of displacement on one of the axes in the focal plane. Near the beam focus, the force is approximately linear with position. We found that for the particle size a distance of 0.45 μm could be used. This criterion is not strictly defined, but a trade off between the signal to noise and the Brownian motion that we know will be measured. The stiffness for large particles increases until peaking for the particle we simulate here. Here, the ‘linear stiffness’, as measured from the peak is about 50% larger than in the shaded region (<450 nm). Even in the region where a linear trend is a good fit, the stiffness varies. Therefore the initial displacement of the particle will always affect the calibration. It will be minimised in the linear fit region and it will very likely lead to increased uncertainty in the calibration. This is approximated in the calculation as a weighted sum for the target stiffness.

A sensitivity analysis of the angular trap frequency could be performed, but, given ample computational resources, it was concluded that the statistics of 1000 simulations would be summarised for relative trap frequency, $\omega_{\mathrm{act}}\Delta t/2\pi =0.1$ with $\Delta t=10^{-4}$ s (sample frequency, $f_{s}=10^{4}$ Hz) and initial starting displacements equal to approximately the beam waist to keep a linear trapping response [1,31,32].

### 2.2. Experiment

Verification of the technique is performed with optical tweezers, consisting of a 1064 nm laser (YLR10-1064-LP, IPG Photonics, Oxford, MA, USA), water immersion objective (UPLSAPO60XW, Olympus Corp, Tokyo, Japan), high-speed camera (EoSens CL MC1392, Mikrotron, Unterschleissheim, Germany) streaming to solid state disk at 10 kfps. Data were post-processed using background image subtraction and several tracking algorithms [33] to establish measurement uncertainty. In order to obtain statistical sampling of the trap stiffness to verify our uncertainty, we use a shutter to simulate the effect of a particle falling into the optical trap. A catch and release experiment was performed on a 2 μm diameter polystyrene particle (No. 19814, Polysciences Inc., Warrington, PA, USA). In thermal equilibrium, the minimum average time that such a particle needs to diffuse a cumulative displacement Δx in 1D is $\Delta t=\Delta x^{2}/2D$. We restrict our interest to an approximately linear trapping region, as shown in Figure 1. We picked a diffusion time of approximately 360 ms followed by a brief time where the beam is turned on to trap the particle for the calibration. This time scale ensures that the particle has sufficient time to travel 400 nm several times over a multi-second sampling time. Approximately one trap period ($2\pi /\omega_{c}$) is required to measure the trap stiffness. Our algorithm selects suitable trapping events, such that the particle diffuses far enough that there will be sufficiently many points to estimate the trap frequency, although not far enough for the non-linear stiffness to affect measurements. As a reference, the particle is recorded undergoing Brownian motion about the trap equilibrium for several seconds, allowing for the trap stiffness to be extracted using power spectral analysis.

## 3. Results

### 3.1. Analysis of Simulation

**Figure 2:**
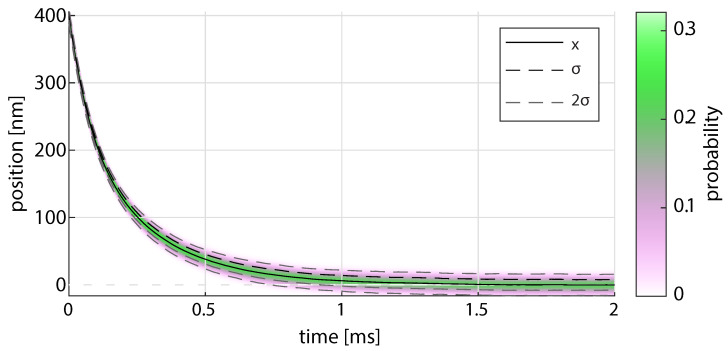
Binned distribution of 1000 simulated sequences of a 1.00 μm radius particles falling into a trap with $\omega_{\mathrm{act}}/2\pi ≃0.1\times 10^{4}$.

Figure 2 shows the binned distribution of the 1000 trajectories from the simulation for the 2 μm particle with the average, standard deviation, and twice the standard deviation. If we consider Figure 1b we can see that the stiffness of the trap is lower at the center of the beam than near the initial fall position. Therefore, fewer points should be included to obtain a calibration characteristic of the initial trapping position. For a wide range of trapping locations, the calibrated stiffness will be somewhere between these two extremes.

**Figure 3:**
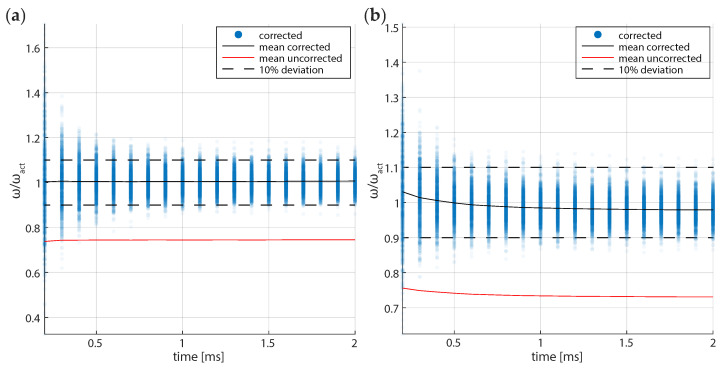
Relative angular trap frequency that is determined by force reconstruction via maximum-likelihood-estimator analysis (FORMA) with sampling time from the initial point. (**a**) Original and modified FORMA computed for an over-damped linear spring model. (**b**) FORMA computed for an accurate optical tweezers toolbox model for the same particle. The discretisation correction to FORMA improves the accuracy of calibration by about 25% for the case that we simulated. Dashed lines of 10% deviation from the simulation parameter value are overlaid as an eye guide.

Our calculations are in a regime where the statistical noise dominates the other two axes. As a result, we use the 1D form of the Maximum Likelihood Esimation (MLE). Using Equation (5) on each of the trajectories, we obtain statistically derived angular trap frequency $\omega_{\mathrm{corr}}/\omega_{\mathrm{act}}=0.98\pm 0.08$ over as little as 2 ms time sequences, as shown in Figure 3. The distribution of the angular trap frequency is approximately Gaussian around the mean and, thus, the uncertainty on a per fall basis is two times the standard deviation. Figure 3a shows the result of FORMA when an over-damped linear spring model is simulated. The mean value othat is btained from calibration is effectively the same as that used to perform the simulation. Figure 3b presents the results for simulations using an accurate optical tweezers toolbox simulation for the same 2 μm particle. Because of the spatially varying stiffness from the model, the trap stiffness will not remain constant. At the level of where the angular trap frequency is $\omega_{c}/2\pi =0.1f_{s}$, we find that the accuracy of the stiffness is improved by about 25% over the uncorrected calibration.

### 3.2. Analysis of Experiments

**Figure 4:**
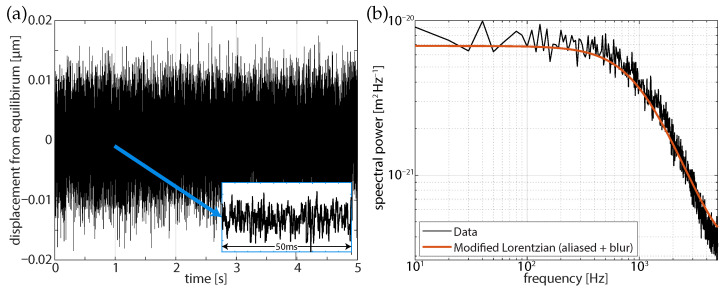
(**a**) Position trace of a 5 s calibration experiment (inset: 50 ms of data at t = 1 s) and (**b**) corresponding power spectrum for 2 μm diameter polystyrene bead, the equilibrium fit yields a stiffness estimate of 118 pN/μ$m^{-1}$ ($\omega_{c}=6942$ s${}^{-1}$) for the optical trap.

The comparison calibration at equilibrium used an aliased power spectrum [5] fit of a 2 μm polystyrene bead as in shown in Figure 4. From the fit Lorentzian we can obtain the optical angular trapping frequency, $\omega_{c}=\frac{k}{\gamma }=2\pi f_{c}$ (trap stiffness, *k*, Stokes drag, γ) which can be used as a reference for further analysis. Here, we fit the power spectrum to Equation (23) of Berg-Sørensen and Flyvbjerg 2004 [5], which incorporates aliasing and blurring and obtain an angular trap frequency of 6942 ± 870 $s^{-1}$, corresponding to a stiffness of approximately 118 pN/μ$m^{-1}$ for the data that are presented in Figure 4. This baseline calibration value can be considered as much as 10% of an underestimate (Figure 1b) of the stiffness, as assessed from a particle falling into the optical trap. Because these data are at equilibrium where blurring is largest, there is no reason to expect that the modified FORMA and power spectrum will agree. Based on the variation of stiffness that is shown in Figure 1b, the fit angular trap frequency could correspond to a predicted angular trap frequency up to about 7636 s${}^{-1}$, where the particle falls into the trap.

**Figure 5:**
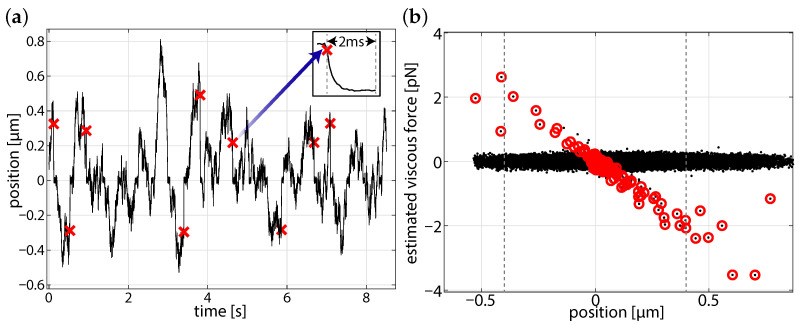
Observations of a 2 μm diameter particle being successively trapped and released. (**a**) Position trace with the start of each in-fall event marked in red. Inset shows a close up an instance of a fall event up to 2 ms after the fall. (**b**) position-force plot with selected in-fall trajectories marked in red dashed gray lines represent the 400 nm maximum starting position.

We require that the particle diffuses sufficiently far from the center of the optical trap, such that there are enough points to reliably determine the angular trap frequency, although not so far that the particle escapes the optical potential. Within a few seconds, several valid trapping events were observed. The start of the selected in-fall events are marked with crosses in Figure 5a. From these events, we are able to construct a position-force curve, where the viscous force has the estimator, $f_{n}=\gamma \frac{y_{n}}{\Delta t}=\gamma \frac{\Delta x}{\Delta t}≃\gamma \dot{x}\left(t\right)$, where γ is the assumed Stokes drag for the particle [14]. 

**Figure 6:**
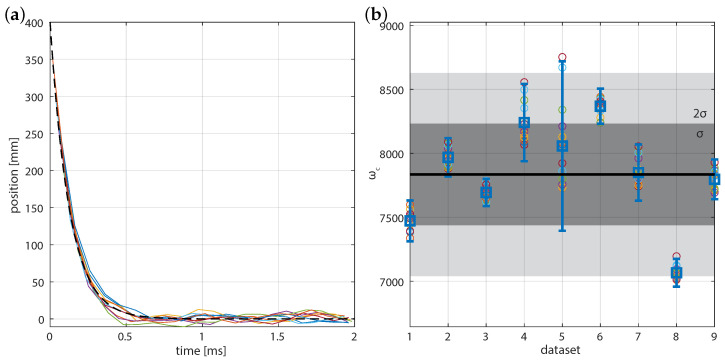
(**a**) Trajectory of in-falling 2 μm particle meeting the criteria that the initial position, $1\geq x_{0}/400\;nm>1/2.5$ to put the data in the high signal-to-noise region. The time traces have been offset in time to a projected 400 nm starting position based on the first measured point. The dashed line is the fall for a particle at the mean angular trap frequency. (**b**) Calculated trap stiffness for several trapping events meeting the criteria for (**a**). The dark and light gray shaded regions respectively represents ±σ and ±2σ of the determined angular trap frequency around the mean.

We expect that for an inertia-less particle in a dispersive system, the particle trajectory in the optical trap will be described by an exponential, $x\left(t\right)=x_{0}e^{-|\omega_{c}|t}$. We can investigate each trajectory to ensure that the particle approaches the expected equilibrium. The overlaid trajectories shown in Figure 6 are windowed series of the in-falls shown in Figure 5a, where we only use positions ≤ 400 nm in our calculation of the optical frequency. This trajectory can also be used to determine the center of the optical trap, by observing the location at which the particle tends toward. Figure 5b demonstrates the improved signal-to-noise of calibration for falling particles when compared to particles near the center of the beam. The flattened distribution near an estimated viscous force of zero is characteristic of the free diffusion between fall events, and there should be no trend between the viscous force and position.

Figure 6 shows multiple trajectories for a single in-falling 2 μm particle, as well as the calculated trap stiffness for several trapping events. We find that, for the data meeting our signal-to-noise rejection criteria (initial fall position, 160 nm$<x_{0}<$ 400 nm) that the angular trap frequency was $\omega_{c}=7834\;\pm \;$ 794 /$s^{-1}$ where the variation in the mean is twice the standard deviation ($2\sigma_{\omega }$) of the value determined from particle tracking criteria (light level threshold) within each 2 ms trajectory (data-set). This compares favorably and with uncertainty of the equilibrium value (∼6900–7600 s${}^{-1}$) taken over 5 s.

## 4. Discussion

### 4.1. Sources of Noise

We encountered low frequency position noise of approximately 8 nm resulting from multiple reflections and air currents distorting the beam and imaging path in the optical system. The noise from the particle tracking algorithm was judged to have a negligible contribution by testing several different pixel rejection criteria leading to root position variances that are typically less than 1 nm.

### 4.2. Trap Linearity with Particle Size and Shape

We have considered particles with a diameter of 2 μm, as these particles are approximately the size of a number of microscopic swimming cells. This size scale is in the geometric optics regime where the object size is larger than the wavelength of light. In these cases, the linear region (optical potential $\propto x^{2}$) could be estimated as the distance just before multiple stable trap locations appear [34] and would scale proportional to particle size. For smaller particles, the maximum displacement for linear response is effectively limited to some fraction of the beam waist. The signal-to-noise of the falling particle in the sub-micron regime will scale proportionally to the square root of particle size. Position only characterisation of the force displacement curve is possible in non-spherical particles, but care must be taken to correctly determine the orientation of the particle [35,36]. In instances of swimming particles, the aim would be to conduct measurements as they swim into the trap. This will be most representative of their hydrodynamic properties.

**Figure 7:**
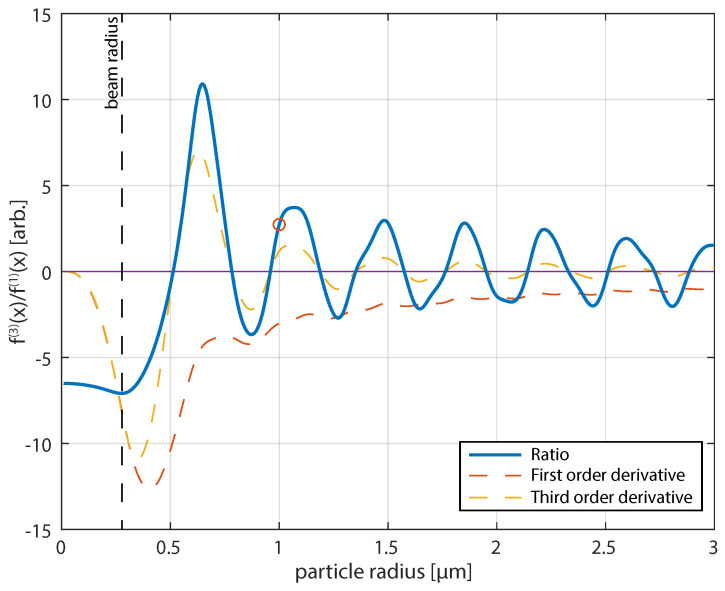
Ratio of first- and third-order derivative as a function of polystyrene particle radius in arbitrary units. The red circle denotes the particle size used in this study. The dashed lines denote the scaled first- and negative third-order derivative.

The rate at which the optical trap begins to deviate from an over-damped harmonic oscillator can be estimated as linear by considering the ratio between the first and third order derivative of the force-extension curve at the beam center. The first order derivative at the beam center will be equivalent to the stiffness of an over-damped harmonic oscillator. The third derivative tells us about how quickly the force-displacement curve deviates. Thus, the ratio of the two gives a relative measure of accuracy that the equilibrium trap frequency can be found from an initial displacement away from the beam center for each particle. Figure 7 shows the ratio of the third and first order derivative of the force-extension curve, the dashed red and yellow lines are the first and negative third order derivatives respectively. All of the curves are scaled to allow comparison. In a Gaussian beam and for fixed laser power, the stiffness of the optical trap scales with the cube of particle size. Because the beam is Gaussian, there is a set relationship between the first and third order derivatives and, thus, the third order derivative scales at the same rate. This changes when the particle is larger than the beam waist. The ratio and hence both equilibrium trap linearity and stiffness begins to oscillate due to the changing optical force contribution of transverse electric and transverse magnetic modes [37].

### 4.3. Observations on Stability

We have mentioned that we are only interested in measurements that lie within the linear region of the position-force curve. We can investigate this requirement in the experimental data by observing the angular trap frequency that is obtained when we vary the number of data points that are used in FORMA and the starting point. We expect that the most reliable results are obtained when the signal-to-noise ratio is largest. Therefore, if we include to many points, or start too close to the equilibrium position, we would expect the calculated frequency to be underestimated since Brownian motion would dominate the trajectory.

**Figure 8:**
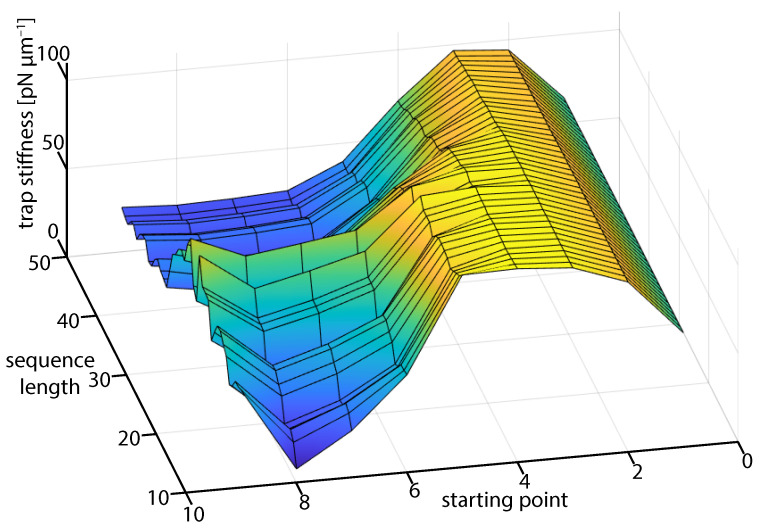
A surface graphic of the trap stiffness determined for particular starting points and duration (sequence length = duration of data capture/Δt) in the particle descent. The stiffness that is determined by FORMA is reduced by blurring of position data obtained near the equilibrium.

From Figure 8, we see that there is a stable region where the trap stiffness changes slowly when we calculate the angular trap frequency using points close to the start of the trajectory, where the particle is travelling at terminal velocity and, hence, has a high signal-to-noise ratio. Although as we take points from further along the trajectory, the blurring and noise in the system begins to dominatem and we can no longer obtain either a statistically meaningful or accurate value for the trap stiffness. This can be understood in terms of the dominating source of information. The information from the initial fall position will improve the overall uncertainty proportional to $1/x_{0}$. At large sample times, the improvement of uncertainty in the equilibrium calibration scales like $1/\sqrt{N_{\mathrm{sample}}}$. The contribution of blurred data to the calibration value at the equilibrium increases as $N_{\mathrm{sample}}$ and deviate the stiffness from the true value. Thus, we have a trade-off between several effects that mean that accurate measurements for a falling particle occur on the order of 1–2 trap periods. Care must be taken to ensure only high signal-to-noise data of sufficient length is used. If the trap has a significant third-order component such as discussed earlier the calibration should be performed as close to the location within the trap that experiments are expected to be performed. This observation is consistent with the simulation where a similar trend was observed (Figure 3) where the estimated stiffness was seen to reduce as more points were included.

## 5. Considerations When Using the Technique

An accurate calibration measurement from the experiment requires that the local stiffness of the optical trap changes weakly as it falls. Particles with radii near the zeros of Figure 7 are ideal for calibration away from the equilibrium. The deviation in the calibration does not significantly improve after a majority of the particle fall is complete and will progressively change as blurring effects becomes a larger portion of the calibration, as discussed in the previous section and with reference to Figure 2a,b. In the theoretical framework we found that the aliasing correction can result in a complex value if too few points are included. This is somewhat ameliorated if the initial fall position is large due to the increase in signal-to-noise.

## 6. Conclusions

We have devised a correction to FORMA, such that a calibration of the trap response can be performed for single trapping events over about ∼2 ms capture time with relative standard deviation, $\sigma_{\omega }/\omega_{c}<6\%$, where various equilibrium determinations will fail due to a combination of low signal-to-noise, blurring, and aliasing. This compared well with a simulated standard deviation of $\sigma_{\omega }/\omega_{c}<5\%$. For the parameters used here in the simulation and experiment (particle diameter, 2 μm, with a NA = 1.2 beam in water), each measurement had a 95% chance of being within 10% of the true value. Two important conclusions for the calibration are: first, the determination of stiffness at equilibrium can lead to significant over- and under-estimates of stiffness with extension, being found from our computational modelling of the force extension curve in Figure 1 and explicit modelling of trap linearity in Figure 7. Second, the calibration is biased toward the first few points under extension at large displacements from the equilibrium due to the increase of optical force over Brownian motion. The equilibrium position can be found by regressing the fall from the calibration to the mean over a short period of time. Our analysis indicates that performing trap calibration as a particle falls will generally reduce the calibration uncertainty and it can be used as an enhancement to standard equilibrium calibration where blurring has been accounted for. Importantly, our results suggest that, in experiments requiring large trap stiffness, such as force-extension experiments in molecular binding assays, microfluidics, or for systems such as biological swimmers, calibration should be performed for large displacements. We see opportunities to develop FORMA to account for blurring effects in the future, but this is likely complicated by use of finite differences increasing the complexity of the de-blurring expression that will be derived. We will use this economical technique in the future to perform rapid particle characterisation of falling particles in microfluidics and for biological swimmers where statistical averaging is typically needed for accurate measurement.

## Data Availability

The data presented in this study are available upon request from the corresponding authors.

## Abbreviations

The following abbreviations are used in this manuscript:

FORMA	Force reconstruction via maximum-likelihood-estimator analysis
MLE	Maximum Likelihood Esimation
